# Transcranial magnetic stimulation therapy for focal leg dystonia: a case report

**DOI:** 10.1186/s40734-019-0076-z

**Published:** 2019-03-08

**Authors:** Kush Sharma, Alberto Cucca, Andrea Lee, Shashank Agarwal, Steven Joel Frucht, Milton Cesar Biagioni

**Affiliations:** 1 The Marlene and Paolo Fresco Institute for Parkinson’s and Movement Disorders, NYU School of Medicine, 222 E 41st Street, 13th Floor, New York, NY 10017 USA; 2 Present address: Parkinson’s Disease & Movement Disorders Institute, at Weill Cornell Medicine, New York, NY USA; 3 Present address: Department of Neurology, at NYU Langone Health, New York, NY USA

**Keywords:** Focal dystonia, Task-specific dystonia, Repetitive transcranial magnetic stimulation, Dystonia therapy

## Abstract

**Background:**

Dystonia is a debilitating disease that causes abnormal, often repetitive, movements, postures or both. The pathophysiology is unknown but related to loss of neuronal inhibition, aberrant sensorimotor integration, and/or derangements of synaptic plasticity. Current treatments include pharmacotherapy, botulinum toxin injections and deep brain stimulation (DBS). The response to these treatments are often limited and carry the risk of side effects requiring alternative therapies such as non-invasive brain stimulation.

**Case presentation:**

We present a case report of a 65-year -old man with refractory focal ‘task-specific’ dystonia. The treatment plan included 10-daily sessions of 1 Hz, 2600 pulses of repetitive transcranial magnetic stimulation (rTMS) targeting the primary motor cortex.

**Conclusion:**

There were no clinical benefits noticed. Currently, there are no rTMS protocol treatments for dystonia. Publication of negative results will help in refining the optimal stimulation parameters, thus maximizing the effectiveness and reproducibility of future therapeutic protocols.

**Electronic supplementary material:**

The online version of this article (10.1186/s40734-019-0076-z) contains supplementary material, which is available to authorized users.

## Background

Dystonia refers to a heterogeneous group of disorders defined as *“*sustained or intermittent muscle contractions causing abnormal, often repetitive, movements, postures, or both”. Dystonic movements are typically patterned and twisting, and may be tremulous. It is often initiated or worsened by voluntary action and associated with overflow muscle activation [1].

The impairment is significantly variable and can range from a mild functional disturbance to complete and severe incapacitating motor dysfunction, resulting in a functional and socioeconomic burden.

The pathophysiology of dystonia is still not clear, but at least three, non-mutually exclusive mechanisms have been proposed: loss of neuronal inhibition, aberrant sensorimotor integration, and/or derangements of synaptic plasticity [2]. A number of neurophysiology studies have documented an abnormal cortical excitability involving the primary motor cortex (M1) of subjects affected by focal and segmental primary dystonia. More specifically, a reduction in intracortical inhibitory mechanisms as defined by reduced short-intracortical inhibition and shortened cortical silent period was extensively reported in the affected motor cortex of these patients, thus supporting the notion that dystonia is likely to be induced by a loss of inhibition of the motor circuitry [3, 4].

Currently, the treatments include pharmacotherapy, Botulinum NeuroToxin (BoNT) injections and deep brain stimulation (DBS). The response to these treatments are often limited and carry the risk of important side effects. For the above reasons, alternative and non-invasive therapeutic strategies are needed.

Transcranial magnetic stimulation (TMS) is a non-invasive brain stimulation (NIBS) technique that can be utilized to probe cortical excitability and perform in-vivo motor brain mapping. TMS pulses can be applied in trains (rTMS) at various frequencies. When used at low frequency, rTMS can generate inhibitory effects, while high frequency rTMS can produce excitatory responses. These effects are often described as LTD- or LTP-like, because the duration of these alterations may implicate changes in synaptic plasticity [5]. rTMS can thus induce long-lasting changes in cortical networks that can be used for therapeutic purposes [6]. Hence, the therapeutic use of low-frequency rTMS in dystonia rests on the rationale that inhibitory TMS could lead to a reduction in the abnormal cortical drive (reducing excitability or plasticity), which would, in turn, decrease aberrant muscular contraction and improve motor function.

### Case presentation

Here, we report a case involving a patient affected by left lower extremity dystonia refractory to BoNT, who was treated with 10 daily sessions of low frequency rTMS at our neurostimulation laboratory to alleviate his symptoms. A 65 year-old male with a history of left leg dystonia for the past three years presented to our clinic for rTMS therapy consultation. The patient is DYT1 mutation negative. The focal dystonia was ‘task specific’ and disabling. While walking forward, posturing was evident in the left lower limb with inversion and plantar flexion of the ankle with hyperextension of the knee (Additional file 1:Video S1). The patient was able to run, walk backwards and go up and down stairs without difficulty. For mobility, he used a non-motorized scooter under the left foot, propelling himself forward with the right foot. There was no clinical evidence of generalized dystonia or parkinsonism. There were also no signs of any psychogenic behaviors such as fixed dystonia, weakness on examination, or a functional etiology. The patient’s past medical and family history was noncontributory. Magnetic resonance imaging of his entire neuro-axis was unremarkable. He initially underwent a trial of carbidopa-levodopa with no benefit. BoNT injections under dual ultrasound and nerve stimulation guidance were performed over two years to a maximum of 500 units, offering no significant improvement in gait. As the patient was considered refractory to conventional treatments and remained functionally impaired, he requested to try rTMS therapy for his FTSD. At the time of evaluation for rTMS, the patient was not taking any medications, and there were no contraindications for the procedure.


Additional file 1:Baseline evaluation of focal leg dystonia before 10 rTMS sessions **Video S1**. (MP4 124671 kb)


**Fig. 1:**
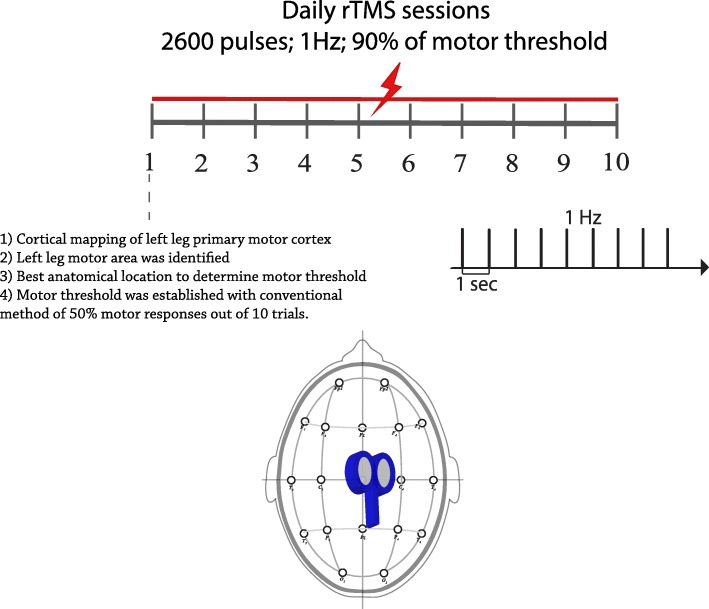
Overview of rTMS procedures

The experimental plan included 10 daily sessions of 1 Hz, low frequency (LF) rTMS targeting the primary motor cortex (left leg motor area of the homunculus). rTMS was delivered using a 70-mm figure-of-8 coil connected to the MAGSTIM Rapid^2^ Stimulator (Magstim, Whitland, Dyfed, UK) with the handle along the sagittal line. A total of 2600 pulses were given as continuous trains for a total of 44 min at an intensity of 90% of his left leg active motor threshold (Fig. 1).

The patient provided written consent for the TMS procedures as well as for the videotaping of the focal dystonia phenomenology. The patient completed self-assessments of Patient Health Questionnaire (PHQ-9), Clinical Global Impression (CGI) and videotaping of unassisted walking at baseline, prior to rTMS, (Additional file 1: Video S1) and follow-up, completion of the ten sessions, (Additional file 2: Video S2).


Additional file 2:Follow up evaluation of focal leg dystonia after 10 rTMS sessions **Video S2**. (MP4 104483 kb)


## Results

The patient tolerated the treatment well and without adverse events. However, after the ten sessions were completed the patient presented with no clear improvement of FTSD, with no change on the PHQ-9 and CGI. Furthermore, there was no apparent change in walking as determined by comparison to the video recordings. A follow-up phone call that took place three weeks post-procedures indicated that there has been no further subjective changes in the patient’s symptoms.

## Discussion and conclusion

Here we report a case of refractory FTSD of the left lower limb showing no clinical benefit from 10 sessions of LF-rTMS over motor cortex. Prior studies utilizing rTMS for the treatment of dystonia have shown modest clinical benefits of rTMS for focal dystonia affecting upper extremities and neck [7, 8]. We conducted an extended rTMS protocol of 10 sessions (the longest reported thus far) with longer session duration (44 min) and marginally lower intensity then prior reports [8, 9] with no evident clinical response. We have chosen to apply an inhibitory rTMS paradigm over patient’s primary motor area based on evidence supporting the role of decreased intracortical inhibition of M1 in the pathophysiology of focal dystonia and restoration of deficient cortico-cortical inhibition after LF-rTMS over M1 [8]. However, alternative targets of stimulation may have resulted in a better clinical outcome. Among these, the premotor cortex (PMC) has been showed to be a suitable target of inhibitory TMS in patients with focal hand dystonia and DYT1 gene carriers. PMC may constitute a more effective target of stimulation by virtue of its widespread bilateral cortical-subcortical connections [10]. This hypothesis, however, remains to be formally tested. Another aspect concerns the duration of treatment. Despite being longer than any prior published studies, we cannot rule out that an even longer treatment plan may have led to a better outcome. Furthermore, the possibility of combining MRI-generated 3D curvilinear reconstruction of the brain along with TMS neuronavigation could have resulted in a more reliable targeting and higher consistency across sessions. Additionally, the utilization of a double-cone coil might have more beneficial for reaching the structures of the lower limbs and the implementation of a properly designed, well-powered, randomized sham-controlled trial is needed to clarify the possibility of this therapeutic tool in this patient population.

The current model according to which low frequency TMS and high frequency TMS are respectively associated to “inhibitory” and “excitatory” effects of the stimulated area is, admittedly, an oversimplification. Neurophysiology of motor cortex was not assessed in this patient to allow a better characterization of the patient’s cortical physiology.

To summarize, here we report a case involving a patient affected by left lower extremity dystonia refractory to BoNT, who was treated with 10 daily sessions of low frequency rTMS with no clinical improvement. This is the first report of rTMS for the treatment of idiopathic adult-onset FTSD of the lower extremities. As optimal protocols for FTSD have not yet been developed. We trust that our contribution will expand the currently limited knowledge regarding the use of rTMS for refractory dystonia. Improving the current publication bias towards negative results will help in refining the optimal stimulation parameters, thus maximizing the effectiveness and reproducibility of future therapeutic protocols. Dystonia pathophysiological models may vary across dystonia subtypes and further studies must take this into account. Individual variability in TMS responses can further result in difficulties validating this method as a therapeutic tool.

## Abbreviations

BoNT: Botulinum neurotoxin
CGI: Clinical Global Impression
DBS: Deep Brain Stimulation
FTSD: Focal Task Specific Dystonia
NIBS: Non-invasive brain stimulation
rTMS: Repetitive transcranial magnetic stimulation

## References

[1] Albanese A, Bhatia K, Bressman SB, Delong MR, Fahn S, Fung VS (2013). Phenomenology and classification of dystonia: a consensus update. Mov Disord.

[2] Quartarone A, Ruge D (2018). How many types of dystonia? Pathophysiological considerations. Front Neurol.

[3] Hallett M (2011). Neurophysiology of dystonia: the role of inhibition. Neurobiol Dis.

[4] Lozeron P, Poujois A, Richard A, Masmoudi S, Meppiel E, Woimant F (2016). Contribution of TMS and rTMS in the understanding of the pathophysiology and in the treatment of dystonia. Front Neural Circuits.

[5] Biagioni MC, Sharma K, Migdadi HA, Cucca A. Non-Invasive Neuromodulation Therapies for Parkinson’s Disease. IntechOpen. 2018. 10.5772/intechopen.75052.

[6] Eldaief MC, Press DZ, Pascual-Leone A (2013). Transcranial magnetic stimulation in neurology: a review of established and prospective applications. Neurol Clin Pract.

[7] Obeso I, Cerasa A, Quattrone A (2015). The effectiveness of transcranial brain stimulation in improving clinical signs of hyperkinetic movement disorders. Front Neurosci.

[8] Siebner HR, Tormos JM, Ceballos-Baumann AO, Auer C, Catala MD, Conrad B (1999). Low-frequency repetitive transcranial magnetic stimulation of the motor cortex in writer's cramp. Neurology..

[9] Kimberley TJ, Borich MR, Arora S, Siebner HR (2013). Multiple sessions of low-frequency repetitive transcranial magnetic stimulation in focal hand dystonia: clinical and physiological effects. Restor Neurol Neurosci.

[10] Huang YZ, Rothwell JC, Lu CS, Wang J, Chen RS (2010). Restoration of motor inhibition through an abnormal premotor-motor connection in dystonia. Mov Disord.

